# *Mycoplasma synoviae *induces upregulation of apoptotic genes, secretion of nitric oxide and appearance of an apoptotic phenotype in infected chicken chondrocytes

**DOI:** 10.1186/1297-9716-43-7

**Published:** 2012-01-26

**Authors:** Daliborka Dusanic, Dusan Bencina, Irena Oven, Ivanka Cizelj, Mojca Bencina, Mojca Narat

**Affiliations:** 1 University of Ljubljana, Department of Animal Science, Chair for Genetics, Animal Biotechnology and Immunology, Groblje 3, 1230 Domzale, Slovenia; 2 National Institute of Chemistry Slovenia, L12 Laboratory of Biotechnology, Hajdrihova 19, 1001 Ljubljana, Slovenia

## Abstract

The role of chondrocytes in the development of infectious arthritis is not well understood. Several examples of mycoplasma-induced arthritis in animals indicate that chondrocytes come into direct contact with bacteria. The objective of this study was to analyze the interaction of an arthrogenic *Mycoplasma synoviae *strain WVU 1853 with chicken chondrocytes. We found that *M. synoviae *significantly reduces chondrocyte respiration. This was accompanied by alterations in chondrocyte morphology, namely cell shrinkage and cytoplasm condensation, as well as nuclear condensation and formation of plasma membrane invaginations containing nuclear material, which appeared to cleave off the cell surface. In concordance with these apoptosis-like events in chondrocytes, transcription was increased in several pro-apoptotic genes. Twenty-four hours after infection, strong upregulation was assayed in *NOS2, Mapk11, CASP8 *and *Casp3 *genes. Twenty-four and 72 h incubation of chondrocytes with *M. synoviae *induced upregulation of *AIFM1, NFκB1, htrA3 *and *BCL2. Casp3 *and *NOS2 *remained upregulated, but upregulation ceased for *Mapk11 *and *CASP8 *genes. Increased production of nitric oxide was also confirmed in cell supernates. The data suggests that chicken chondrocytes infected with *M. synoviae *die by apoptosis involving production of nitric oxide, caspase 3 activation and mitochondrial inactivation. The results of this study show for the first time that mycoplasmas could cause chondrocyte apoptosis. This could contribute to tissue destruction and influence the development of arthritic conditions. Hence, the study gives new insights into the role of mycoplasma infection on chondrocyte biology and development of infectious arthritis in chickens and potentially in humans.

## Introduction

Species from the genus *Mycoplasma *have long been recognized as important pathogens that cause diseases of the respiratory tract, urogenital tract and joints in a variety of animal species, including humans. *Mycoplasma synoviae *(*M. synoviae*) is a common pathogen of chickens and turkeys. It usually colonizes the respiratory tract, although this condition can lead to the development of systemic infection and/or infectious synovitis [1-4]. Experimental infectious synovitis has been associated with the hemagglutination-positive phenotype of *M. synoviae *[5]. The condition is presented as an acute joint swelling accompanied by increased volume of synovial fluid, infiltration with macrophages, B and T cells, lining cell hyperplasia and fibroblastic proliferation. In the acute phase of infection, live *M. synoviae *have been detected in synovial fluids of infected birds, suggesting that *M. synoviae *can come into direct contact with chondrocytes as is the case with *M. pulmonis *[6] and *M. artritidis *[7]. *M. synoviae *has been reported capable of invasion into non-phagocytic chicken cells including chondrocytes *in vitro *[8].

Mycoplasmas have been reported in many studies as active players in host-pathogen interactions leading to alterations in cell death patterns (Table 1) [9-22]. To our knowledge, there has been no report of any *Mycoplasma *species having apoptosis modulating effects on infected chondrocytes. Therefore, we conducted this study to elucidate the impact of *Mycoplasma synoviae *infection on chicken chondrocytes.

**Table 1:** *Mycoplasma *species with reported roles in apoptosis modulation.

*Mycoplasma *species	Host species	Disease(s)	Apoptosis modulation mechanism	Reference
*M. hyorhinis*	swine	Polyserositis, otitis media, arthritis, pneumonia	Induces apoptosis in human carcinoma cell line AZ-521 by activation of NOS2 and caspase 3.	[9]

*M. bovis*	cattle	Chronic unresponsive pneumonia, systemic infection, mastitis, arthritis	Inhibits proliferation without apoptosis or effect on function of lymphocytes and peripheral blood mononuclear cells. Induces apoptosis of bovine lymphocytes.	[10,11]

*M. fermentans*	humans, mice	Possible role in arthritis (found in patients)	Reduces TNF-induced apoptosis upstream of caspase 8 in a myelomonocitic cell line. Prevents apoptosis of 32D cells by inducing *NFκB *gene upregulation. Promotes immortalization of human peripheral blood mononuclear cells in culture. Promotes proliferation of synovial fibroblasts.	[12-15]

*M. alligatoris*	alligators	Acute lethal cardiopulmonary disease	Promotes *CD95 *expression and apoptosis of primary cardiac fibroblasts through modulation of CD44 receptor.	[16]

*M. hominis*	humans	Possible role in arthritis (found in patients)	Induces cell death in HeLa cells (through ATP release) and in 32D cells.	[14,17]

*M. penetrans*	humans	Possible role in arthritis (found in patients)	Mediates NFκB activation in mouse macrophages and induces apoptosis. Prevents apoptosis of 32D cells by inducing NFκB.	[14,18]

*M. arginini*	humans, rodents, camels, sheep, pigs	Pneumonia, chronic respiratory disease, otitis	Inhibits apoptosis of cells expressing toll-like receptors 2/6 by inducing constitutive NFkB activation and p53 suppression. Suppression of *tp53 *gene induces carcinogenic and mutagenic effects on rodent fibroblasts.	[19,20]

*M. genitalium*	humans	Urethritis, pelvic inflammatory disease, pneumonia, arthritis	Does not affect 32D cells. Induces apoptosis in human monocytes THP-1 by activating NFκB.	[14,21]

*M. pneumoniae*	humans	Atypical pneumonia, possible role in arthritis	Supports continous growth of 32D cells.	[22]

Apoptosis is a highly regulated process of cell demise and is generally induced through activation of different components of two overlapping signaling pathways. The extrinsic pathway includes the activation of death receptors leading to activation of a family of cysteine proteases known as caspases [23]. Proteolytic cleavage of around 400 caspase substrates that have been identified so far [24] leads to phenotypic features of apoptosis. These include rounded morphology, condensed cytoplasm, fragmented organelles and formation of plasma membrane blebs that are separated from the cell into independent vesicles known as apoptotic bodies (reviewed in [25]). The intrinsic pathway is linked to stress signals from within the cell. It involves cleavage of Bid into tBid leading to activation of Bax and Bak1. They form a pore in the outer mitochondrial membrane and cause the release of proteins from the mitochondrial intermembrane space (endonuclease G, AIFM1, cytochrome c etc.) leading to execution of either caspase independent DNA cleavage (AIFM1, endonuclease G) [26] or the formation of apoptosome and activation of caspase 3 [27,28].

One of the key players in the induction of apoptosis of articular chondrocytes in humans is nitric oxide (NO), which is produced by immune cells in the synovium and pannus as well as by activated chondrocytes [29-32]. NO induces apoptosis through p38 MAP kinase (MAPK11) mediated stimulation of caspase 3 activity and accumulation of nuclear factor kB (NFkB). This leads to increased expression of p53, a signaling molecule that acts upstream of caspase 3 [33,34], as well as upregulation of pro-apoptotic Bax. Another mechanism of apoptosis in arthritic cartilage includes activation of CD95 (Fas) death receptor leading to upregulation of Fas ligand and p38 MAP kinase [35].

In this study, *M. synoviae *infected chicken chondrocytes (CCH) were evaluated during three days for changes in cell viability, morphology and expression of genes associated with apoptosis.

## Materials and methods

### CCH cell culture

Chicken chondrocytes (CCH) were obtained from hyaline cartilage of hock joints as described previously [8]. Primary cultures of CCH were cultivated up to the sixth passage in Dulbecco Modified Eagles Medium (DMEM), supplemented with 7.5% fetal bovine serum (FBS) and 2.5% chicken serum (all from Sigma-Aldrich, Munich, Germany). Cells were incubated at 37°C in a 5% CO_2 _incubator.

### Jurkat cell culture

Jurkat cells (ATCC, CCL TIB 152, kindly donated by Prof Nataša Kopitar Jerala, Jožef Stefan Institute, Slovenia) that overexpress death receptors were used as a positive control for apoptosis. Cells were cultivated in DMEM supplemented with 10% FBS and incubated at 37°C in a 5% CO_2 _incubator.

### *Mycoplasma synoviae *cultures

Cultures of *M. synoviae *type strain WVU 1853 (approximately 20 in vitro passages before being used in this study) were grown in Frey mycoplasma broth as described previously [8]. Bacteria in the logarithmic phase of growth were used for CCH infection. The number of colony forming units (CFU) was determined as described previously [36].

### XTT-based cell viability test

To perform the cell viability test, CCH were seeded into 96 well plates (Corning, New York, USA) at density 5 × 10^5 ^cells/mL and incubated overnight at 37°C in a 5% CO_2 _incubator prior to infection. *M. synoviae *broth culture in the logarithmic phase of growth was used to infect CCH in a ratio of 10-100 *M. synoviae *CFU per CCH. As a positive control of apoptosis, 5-fluorouracil was used (5-FU, final concentration 20 μg/mL, from Sigma-Aldrich, Munich, Germany). Non-infected CCH were used as negative controls. As additional controls of the experiment, Jurkat cells, seeded at density 5 × 10^5^/well, were subjected to the same three procedures. Tetrazolium-based dye XTT (Sigma-Aldrich, Munich, Germany) was added to wells after 0, 24, 48, 72 and 96 h of incubation. The final concentration of XTT in cell suspensions was 20%. Following 3 h of incubation at 37°C in a 5% CO_2 _incubator, absorbance of the samples was measured at 492 nm. The background signal was measured at 690 nm. The test was performed in three independent experiments with triplicates of each experimental condition. Average absorbances were calculated using MS Excel. Student *t*-test for independent values was used for determining statistically significant differences (*p *< 0.05).

### Phase contrast and confocal fluorescence microscopy

Morphological changes were observed by phase contrast and confocal fluorescence microscopy. CCH were seeded onto cover slides at density 5 × 10^5 ^cells/mL and incubated overnight. The cells were then infected with *M. synoviae *as described and incubated for 24 h, 48 h and 72 h followed by phase contrast microscopy examinations for morphological changes. Additionally, distribution of CD44 receptor (hyaluronan receptor; its cross-linking augments Fas expression and subsequent Fas-mediated apoptosis of the cells [37]), and nuclear condensation were observed using confocal fluorescence microscopy. The medium was aspirated, and CCH were washed in sterile phosphate buffered saline (PBS). The cells were fixed using a pre-cooled mixture of acetone and methanol (both from Sigma-Aldrich, Munich, Germany) for 10 min at room temperature. A solution of 10% goat serum (Sigma-Aldrich, Munich, Germany) was used for blocking (1 h, room temperature), followed by 3 washes in PBS containing 0.5 mg/mL RNase (Sigma-Aldrich, Munich, Germany). Mouse monoclonal antibodies to chicken CD44 (DSHB, Iowa City, USA), diluted 1:100 in PBS were added for 1 h at room temperature. After washing, Alexafluor-488 goat anti-mouse IgG antibodies, diluted 1:2000 (Life Technologies - Invitrogen, Carlsbad, USA), were used as secondary antibodies. After 1 h at 37°C, unbound antibodies were washed off, and nuclei were labeled for 5 min in propidium iodide (Sigma-Aldrich, Munich, Germany) in PBS with 0.1% Triton X-100 and 0.5 mg/mL RNase (both from Sigma-Aldrich, Munich, Germany). Following washing in PBS, cover slides were mounted using Dako Fluorescence Mounting medium (Dako, Glostrup, Denmark) and viewed using a Leica TCS SP5 confocal microscope (Leica Microsystems, Mannheim, Germany).

### RT-qPCR

In order to perform gene expression analysis, CCH were seeded into 75 cm^2 ^flasks (Techno Plastic Products, St. Louis, USA) at density 5 × 10^5 ^cells/10 mL and incubated overnight. The cells were then treated as described for the XTT assay and sampled by trypsinization after 24, 48 and 72 h of incubation. CCH were washed in sterile PBS followed by isolation of total RNA using RNeasy Mini Kit (Qiagen GmbH, Hilden, Germany). RNA concentration was determined spectrophotometrically using NanoVue (GE Healthcare, Waukesha, USA). Subsequently, RNA was stored at -80°C. Residual DNA was eliminated by incubating 1 μg of RNA with 1U of RNase-free DNase I in a buffer containing MgCl_2 _ions (both from Thermo Fisher Scientific - Fermentas, St. Leon-Rot, Germany) for 30 min at 37°C. Following DNase inactivation at 65°C, cDNA was synthesized using a High-Capacity cDNA Reverse Transcription Kit (Life Technologies - Applied Biosystems, Foster City, USA), according to the manufacturer's instructions. cDNA was stored at -20°C immediately after reverse transcription reactions. Prior to use, primer pairs were checked for specificity in silico using NCBI Primer BLAST [38]. Primer efficiency was checked on each primer pair using dilutions of cDNA combined from all experimental conditions and timepoints.

For RT-qPCR reactions, 20 μL mixtures were made, containing 10 μL of Power SYBR Green PCR Master Mix (Life Technologies - Applied Biosystems, Foster City, USA), 0.5 μL of forward and 0.5 μL of reverse primer (final concentration 5 μM, Integrated DNA Technologies, Leuven, Belgium), 8 μL of DEPC-treated water (Qiagen GmbH, Hilden, Germany) and 1 μL of cDNA. Stratagene Mx3000P (Agilent Technologies - Stratagene, Santa Clara, USA) was used to perform RT-qPCR reactions and its MxPro software for analysis of amplification and dissociation plots. Reaction conditions were set to 10 min at 95°C (first segment, one cycle), 15 s at 95°C and 1 min at Tm of a specific primer pair (second segment, 40 cycles) followed by one cycle with 15 s at 95°C, 30 s at Tm and 15 s at 95°C (dissociation curve segment). Gene expression was analyzed for 15 genes (Table 2, [39]), and *GAPDH *was used as a reference gene [40]. Gene expression values of non-infected CCH were used for gene expression calibration. Appropriate controls (no template and no reverse transcription control) were also performed in each run.

**Table 2:** A list of oligonucleotides used as primers in RT-qPCR analysis of gene expression in chicken chondrocytes.

Gene symbol	RefSeq mRNA number	Forward primers	Reverse primers	Amplicon lenght	Reference	Encoded protein function
*Casp3*	NM_204725.1	TGGCCCTCTTGAACTGAAAG	TCCACTGTCTGCTTCAATACC	139	IDT^1^	**Caspase 3**: apoptosis related cysteine peptidase from the group of effector caspases.

*Bak1*	NM_001030920.1	GCCCTGCTGGGTTTCGGTAA	AATTCGGTGACGTAGCGGGC	95	NCBI^2^	**Bcl2 antagonist/killer**: pro-apoptotic member of the Bcl2 family, involved in mitochondrial pore formation.

*Fas*	XM_421659.2	CCTGCTCCTCATCATTGTGT	TGATCCATGTACTCCTCTCC	258	[20]	**Fas**: TNF receptor superfamily, member 6.

*FASLG*	NM_001031559.1	GGAGAAGGAACTGGCTGAAC	GGTTTCCTGTTAAGTGTGCTG	134	IDT^1^	**Fas ligand**: TNF superfamily, member 6.

*tp53*	NM_205264.1	ACCTGCACTTACTCCCCGGT	TCTTATAGACGGCCACGGCG	127	NCBI^2^	**Tumor protein p53**: tumor suppressor, activator of apoptosis through induction of BAX expression.

*AIFM1*	NM_001007490.1	GAAGTACAACAACGGCTGAC	GAGACAGAGACAGACTTGAC	299	IDT^1^	**Apoptosis-inducing factor**: pro-apoptotic mitochondrial protein, responsible for caspase-independent DNA cleavage.

*CASP8*	NM_204592.2	GGACAGGACTGAGCTGGCGT	AGGTCCCCCACCTCGATCATC	135	NCBI^2^	**Caspase 8**: apoptosis related cysteine peptidase from the group of initiator caspases.

*BCL2*	NM_205339	GATGACCGAGTACCTGAACC	CAGGAGAAATCGAACAAAGGC	114	IDT^1^	**B-cell CLL/lymphoma 2**: anti-apoptotic member of the Bcl2 family, blocks mitochondrial pore formation.

*endog*	XM_415487.2	GAACAACGTGGCCGTCCCT	GGGCATCACATAGGAGCGCA	91	NCBI^2^	**Endonuclease G: **mitochondrial endonuclease, released from mitochondria during apoptosis.

*XIAP*	NM_204588.1	GCAGAATATGAGAGGCGGATAC	TCCTTCCACTCTTGCAATCC	149	IDT^1^	**X-linked inhibitor of apoptosis: a**nti-apoptotic protein from the IAP family, blocks caspase 3 activation by inhibiting caspase 9.

*NOS2*	NM_204961.1	GCATTCTTATTGGCCCAGGA	CATAGAGACGCTGCTGCCAG	66	IDT^1^	**Nitric oxide synthase 2, inducible**: pleiotropic immune system regulator, inducer of apoptosis in chondrocytes.

*NFκB1*	NM_205134.1	AGGACTTAAAATGGCAGGAGAG	GCTGTTCGTAGTGGTAAGTCTG	141	IDT^1^	**Nuclear factor of kappa light polypeptide gene enhancer in B-cells 1**: transcription factor with pro- or anti-apoptotic functions.

*CD44*	NM_204860.2	AGCACTGGTCTTTACTGGAAC	TCTGAGCAACTTGGGAAACTG	93	IDT^1^	**CD44 molecule (Indian blood group)**: hyaluronan receptor, involved in interaction of the cell with the extracellular matrix, capable of inducing caspase-independent death via calpain-dependent AIF release. Crosslinking of CD44 augments Fas expression and mRNA transcription.

*htrA3*	XM_420813.2	CCTCCCGCGGCTTCGTATTC	TGCAGTCGCGGTGCAGTAAG	116	NCBI^2^	**HtrA serine peptidase 3**: mitochondrial serine peptidase, inhibits XIAP.

*Mapk11 (p38B)*	NM_001006227.1	GCGGCTCCGCTAAAATGCCG	GGGGTGAGGTTCTGGTAGCGC	97	NCBI^2^	**Mitogen-activated protein kinase 11**: stimulates caspase 3 activity, *NFκB *expression and *p53 *expression and protein stability.

*GAPDH*	NM_204305.1	ATGGCATCCAAGGAGTGAGC	AACAAAGGGTCCTGCTTCCC	66	[39]	**Glyceraldehide-3-phosphate dehydrogenase**: enzyme in carbohydrate metabolism.

^1 ^Designed in Integrated DNA Technologies PrimerQuest^SM ^application ^2 ^Designed in NCBI Primer-BLAST application

Three independent experiments were performed to collect RNA for RT-qPCR. Relative gene expression was assayed in each experiment and experimental condition separately. Three repeats of each RT-qPCR reaction were performed. Normalized relative quantities were calculated using the efficiency corrected 2^-ΔΔCq ^method [41]. The effect of intra-assay variation on the statistical significance of the results was reduced by log transformation of normalized relative quantities, mean centering and autoscaling as described in [42]. Statistical significance of the results was determined using the Student *t*-test (*p *< 0.05) and statistically significant results visualized through Ingenuity Pathways Analysis platform (Ingenuity Systems, Redwood City, USA).

### Nitric oxide assay

In order to determine the concentration of NO, an experiment equal to XTT-based cell proliferation test was set up using CCH. After 24, 48 and 72 h of incubation, supernates were transferred to a sterile 96-well plate and assayed immediately using the Griess assay (R&D Systems, Minneapolis, USA). The concentration of NO was determined based on the standard curve and absorbances of the azo-compound NO forms in the reaction with sulfanilamide and N-1-naftyletylendiamin dihydrochloride. Absorbances were read at 550 nm (background at 630 nm). Two independent experiments with three replicates of each condition were performed and mean absorbances ± standard errors were used to test statistical significance of the results (Student *t*-test, *p *< 0.05).

## Results

### *M. synoviae *decreased CCH and Jurkat cell respiration (viability)

**Figure 1:**
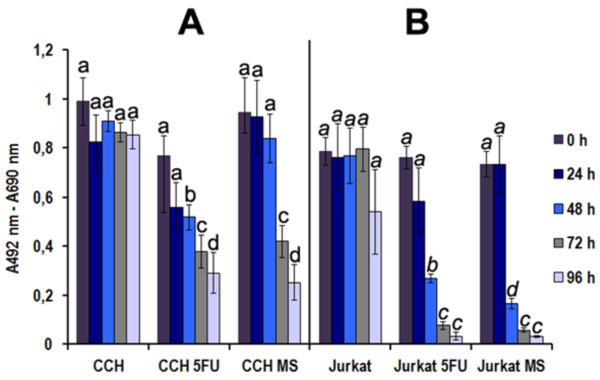
**Cell respiration (viability) of control cells, cells treated with 5-fluorouracil (5-FU) or infected with *M. synoviae***. CCH or Jurkat cells were treated for indicated time periods and assayed using the XTT-based cell viability test. The results are presented as mean values ± standard error for three independent experiments with three replicates each. Means with a different letter are different (*p *< 0.05 to *p *< 0.001) by the Student *t*-test.

We assayed the respiration of CCH by determining the relative decrease in reduction of tetrazolium salt XTT in *M. synoviae *infected CCH compared to the negative control. As a positive control, CCH were treated with 5-FU. Jurkat cells were used as an additional positive control. *M. synoviae *infection caused significant decrease in CCH respiration, indicating reduced viability. This effect was noticed 72 h and 96 h after infection (*p *≤ 0.1) (Figure 1). An even stronger decrease in respiration, already after 24 h, was noticed in CCH treated with 5-FU (Figure 1). Jurkat cells responded stronger than CCH to both treatments, with a major decrease in cell respiration apparent already after 24 h in both cases (*p *< 0.01) (Figure 1). Fresh medium was not added to any of the cells at the time of measurements, resulting in a decrease in viability of fast-growing Jurkat cells even in negative controls after 96 h (Figure 1).

### *M. synoviae *infected CCH exhibited apoptotic morphology and nuclear condensation

**Figure 2:**
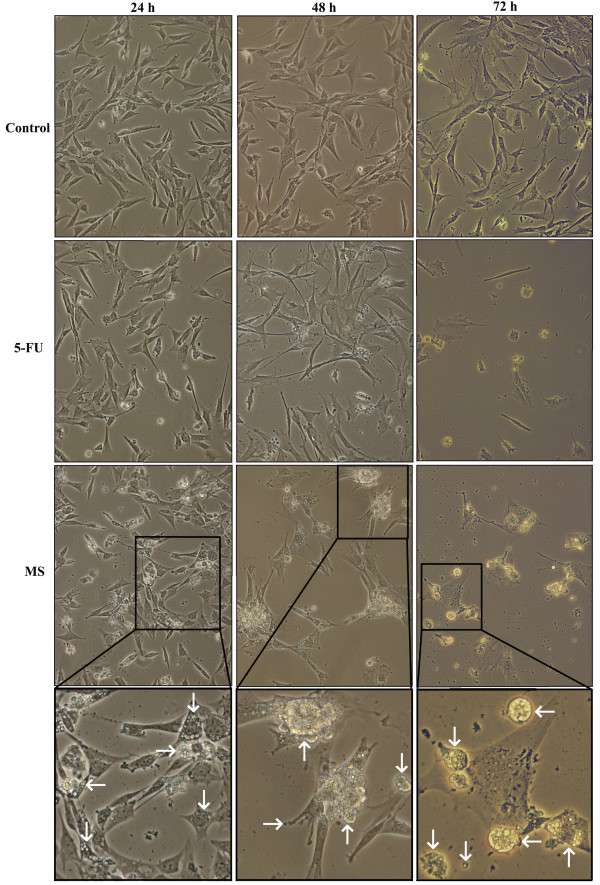
**Cytopathological changes in control CCH, CCH treated with 5-fluorouracil (5-FU) or infected with *M. synoviae***. CCH were seeded onto cover slides and incubated overnight followed by treatment with 5-FU or infection with *M. synoviae *and incubation for 24 h, 48 h and 72 h. In each timepoint, cells were observed for morphological changes by phase contrast microscopy (400 × magnification). Typical examples of chondrocyte morphology after infection with *M. synoviae *are shown in magnification. Vacuolization and indications of membrane blebbing are indicated by arrows.

Phase contrast microscopy of infected CCH revealed dramatic changes in cell morphology (Figure 2). Twenty-four hours after infection with *M. synoviae*, some CCH were shrinking. Plasma membranes appear rough but formation of membrane vesicles was not noticed. The cytoplasm of infected cells shows increased vacuolization. However, the number of cells that adhered to cover slides was roughly comparable to the control (Figure 2). Longer exposure to *M. synoviae *resulted in more pronounced vacuolization of cytoplasm and formation of membrane blebs seen 48 h after infection (Figure 2). After 72 h, cytoplasm vacuolization was present in some of the uninfected CCH, to which no fresh medium was added after seeding into plates. Still, most cells were attached to cover slides. In contrast, only a few cells infected with *M. synoviae *remained attached to cover slides (Figure 2). The remaining ones exhibited excessive membrane blebbing with individual vesicles detaching from cells or cytoplasm condensation (cell shrinkage) and/or vacuolization (Figure 2). In CCH treated with 5-FU, membrane roughness and cell shrinkage was noticed after 24 h, but with no cytoplasm vacuolization present. A similar morphology was observed 48 h after treatment, but in a larger number of cells. The number of cells still attached to the surface was lower than in the control slide and some cells presented with membrane blebs. Seventy-two hours of treatment with 5-FU caused cell death and detachment from cover slides in most cells, with remaining cells presenting similar morphological features as those infected with *M. synoviae *(Figure 2).

**Figure 3:**
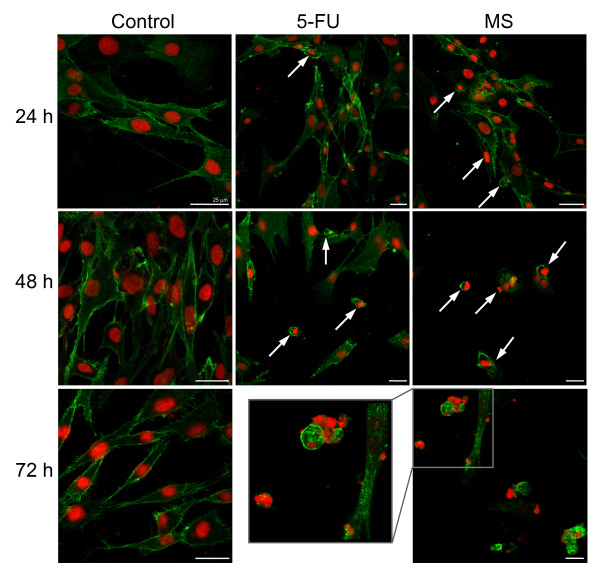
**Morphological and other changes in untreated CCH, CCH treated with 5-fluorouracil (5-FU) or infected with *M. synoviae***. CCH were seeded onto cover slides and incubated overnight. Cells were then treated with 5-FU or infected with *M. synoviae *and incubated for 24 h, 48 h and 72 h followed by fixation in an acetone-methanol mixture, blocking and labeling with mouse antibodies to chicken CD44. Alexafluor-488 goat antibodies to mouse IgG were used as secondary antibodies and nuclei were labeled with propidium iodide. Nuclear condensation and formation of blebs containing nuclear material are indicated by arrows.

Confocal microscopy of infected CCH labeled with antibodies to membrane receptor CD44 and propidium iodide revealed additional changes caused by *M. synoviae *infection (Figure 3). The nuclei of negative controls stained evenly with PI after all three timepoints, while brighter foci indicating condensed DNA were present in CCH infected with *M. synoviae*. Additionally, vesicles detaching from the blebbing CCH plasma membrane contained condensed DNA. Differences in CD44 receptor abundance and distribution were not obvious in CCH infected with *M. synoviae*. CCH treated with 5-FU appeared to express more CD44 (Figure 3).

### *M. synoviae *induced upregulation of pro-apoptotic genes

**Figure 4:**
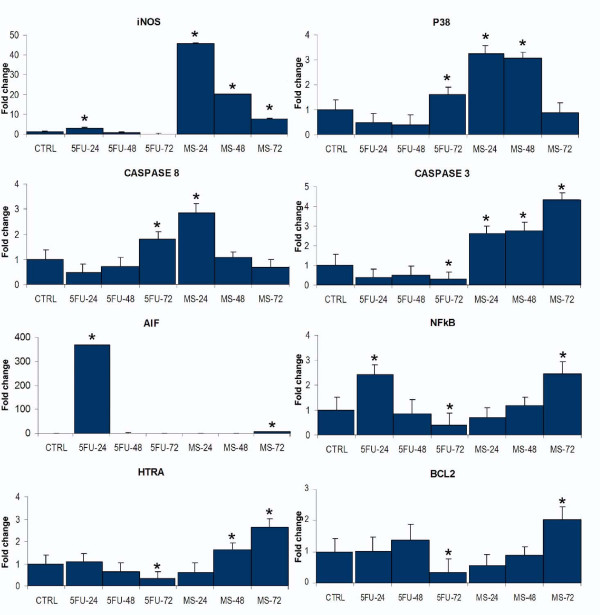
**Expression of selected genes after exposure of CCH to 5-fluorouracil or *M. synoviae *WVU 1853**. Exposure agent and time of exposure (in hours) are indicated below columns. Control (CTRL) represents in all graphs non-exposed CCH. The results are given as mean values ± standard error for three independent cell treatment experiments with three RT-qPCR replicates for each experiment. Means marked with stars are significantly different from controls (*p *< 0.05 to *p *< 0.001) by Student *t*-test.

Expression of 15 genes (Table 2) was assayed in CCH exposed to 5-FU or infected with *M. synoviae*. The level of expression was normalized to untreated control cells and calibrated with reference to *GAPDH *expression. Upregulation of several genes was already observed in the cells sampled 24 h after infection. The gene encoding inducible nitric oxide synthase (*NOS2*) was upregulated 46-fold (*p *< 0.001), *Casp3 *was upregulated 2.6-fold (*p *< 0.001), *Mapk11 *3.2-fold (*p *< 0.01) and *CASP8 *2.9-fold (*p *< 0.01) (Figure 4). In CCH infected with *M. synoviae *for 48 h, *Casp3, NOS2 *and *Mapk11 *remained upregulated (2.8-fold, 20.1-fold and 3.1-fold, respectively, *p *< 0.001), while another gene, *htrA3*, became slightly upregulated (1.6-fold, *p *< 0.01) (Figure 4). Seventy-two hours after infection, *Casp3 *and *NOS2 *remained upregulated (4.4-fold and 7.7-fold, respectively, *p *< 0.001), while *Mapk11 *was no longer upregulated. A strong increase in transcription was noticed in genes *AIFM1 *(6.7-fold, *p *< 0.001), *NFκB1 *(2.5-fold, *p *< 0.001) and *htrA3 *(2.7-fold, *p *< 0.001). *BCL2 *was also upregulated (2-fold, *p *< 0.001) (Figure 4). No change in gene expression at all tested timepoints was noticed for *Bak1, endog, tp53, CD44, XIAP, Fas *and *FASLG *(Additional file 1, Figure S1).

CCH treated with 5-FU revealed a pattern of gene expression that differed considerably from that of CCH infected with *M. synoviae*. As indicated by the XTT test and morphological changes, the effect of 5-FU was more pronounced compared to *M. synoviae*, especially 24 and 48 h after treatment. After 24 h, strong upregulation was noticed in *CD44 *(11.3-fold, *p *< 0.001), *AIFM1 *(369.2-fold, 0.001), *Fas *(11.9-fold, *p *< 0.001), *FASLG *(4.3-fold, *p *< 0.05), *NOS2 *(3.1-fold, *p *< 0.01), and *NFκB1 *(2.4-fold, *p *< 0.01). Interestingly, the expression of *XIAP *was also elevated after 24 h (3.6-fold, *p *< 0.01). Increasing the incubation time to 48 h revealed a normalization of *NFκB1, XIAP, AIFM1 *and *NOS2 *expression together with upregulation of *tp53 *(1.8-fold, *p *< 0.05) and lower level of upregulation in *CD44, Fas *and *FASLG *(Figure 4). Seventy-two hours of incubation of CCH with 5-FU-supplemented medium left little living cells, in concordance with the results of the proliferation test and morphological observations, and these had only a few upregulated genes, including *Fas *(1.5-fold, *p *< 0.05), *Bak1 *(1.3-fold, *p *< 0.05), *CASP8 *(1.8-fold, *p *< 0.001) and *Mapk11 *(1.6-fold, *p *< 0.01) (Figure 4, Additional file 1, Figure S1).

**Figure 5:**
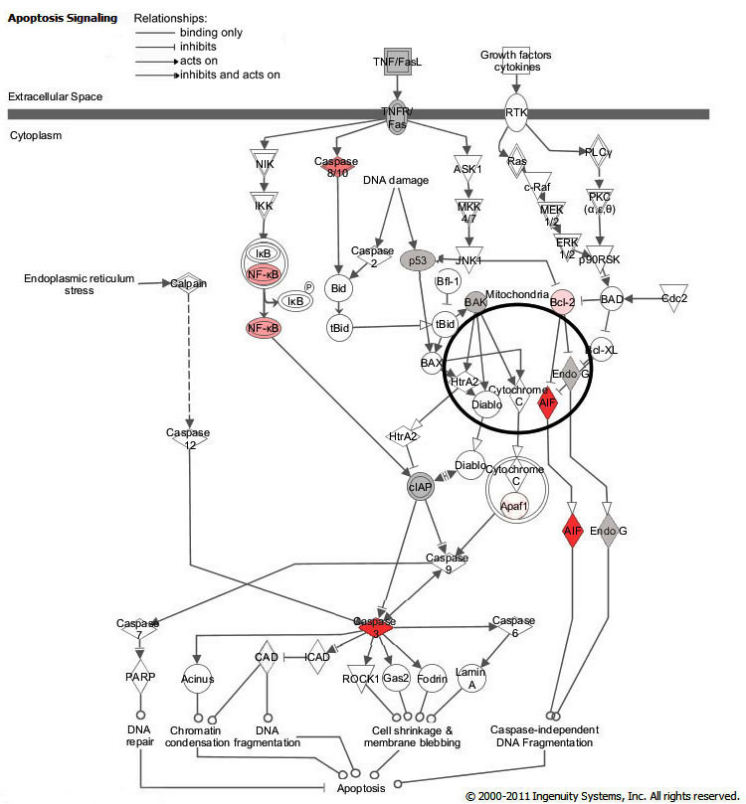
**Ingenuity Pathway Analysis network depicting interactions between genes known to be involved in apoptosis induction**. Two major pathways are shown, the death receptor (extrinsic) pathway, and mitochondria-dependent (intrinsic) pathway. Pink color of molecules indicates upregulation of genes analyzed in this study (level of upregulation after 24, 48 or 72 h of CCH exposure to *M. synoviae*), with darker shades of pink representing higher levels of gene upregulation. Grey color indicates no change in gene expression, whereas molecules that were not analyzed are white. Types of relationships are explained in the legend. The pathways depicted represent conserved pathways generated from knowledge published for different species. (Note that exceptions specific for avian species could be possible).

Interactions between proteins encoded by genes of interest, as well as levels of gene upregulation, were depicted using the Ingenuity Pathways platform (Figure 5).

### Chondrocytes infected with *M. synoviae *produced nitric oxide

**Figure 6:**
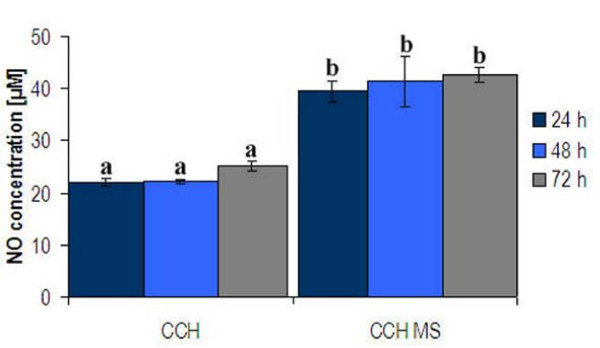
**Concentration of NO in culture media of CCH infected with *M. synoviae *for 24, 48 and 72 h**. NO concentration was determined by the Griess assay. The results are presented as mean concentrations ± standard error for two independent experiments with three replicates each. Different letters above columns indicate significant differences between NO concentrations (Student *t*-test, *p *≤ 0.01).

Infection of CCH with *M. synoviae *induced a significant increase in NO production at all tested time points (*p *≤ 0.01) (Figure 6), in agreement with the strong upregulation of *NOS2*. NO concentration doubled already 24 h after infection. During the next 48 h, the concentration of NO did not increase significantly compared to the concentrations assayed after 24 h.

## Discussion

In the research of rheumatoid arthritis etiology, the idea of a bacterial infection leading to arthritis is becoming increasingly popular [43-45]. Bacterial infections increase the production of NO, and can lead to inflammation. Inflammation then leads to chondrocyte apoptosis [30-35]. In cartilage tissue that is rich in extracellular matrix and with few cells in hard to reach places, clearance of dying cells is especially troublesome and can lead to the development and perpetuation of inflammation, which in turn leads to cartilage and bone degradation [46-48]. The idea of mycoplasmal infections, as the triggering factor of rheumatoid arthritis, has been revisited several times. Still, the role of mycoplasmas that have been detected in synovial fluids and serums of patients suffering from arthropathies remains unclear [49-54]. Factors impeding these studies are many. Because of their obligatory parasitism and complex nutritional demands, mycoplasmas are hard to cultivate. In addition, mycoplasmas adapt to the host in a way that induces subclinical infections that pass with little or no inflammation. Furthermore, mycoplasmal antigens, many of which are enzymes, can affect cells even after bacteria are dead and lysed, and their presence is difficult to detect by certain conventional methods [49]. *Mycoplasma *species are also well known to cause arthritis in certain animal species, including chickens [1-7]. Experimental infections of chickens with *M. synoviae *WVU 1853 resulted in thinning of the articular cartilage 25 days post infection [55]. Visible cartilage erosion appeared about two weeks later, presumably due to the presence of increasing numbers of heterophils. It seems likely that *M. synoviae *could also affect CCH. This assumption is supported by our observations that synovial fluids of chickens infected experimentally into hock joints contained antibodies to CCH and cartilage proteins (Dušanić et al., unpublished observations). This suggests CCH death and tissue destruction, leading to consequent production of autoantibodies against cartilage and CCH proteins. Many *Mycoplasma *species invade non-phagocytic cells and interfere with apoptosis (Table 1) [9-22]. *M. synoviae *is capable of invading CCH [8]. The interactions between CCH and *M. synoviae *are likely to occur via binding of *M. synoviae *hemagglutinin VlhA to CCH receptors containing Sia (α 2-6) gal and Sia (α 2-3) gal, and their desialylation with *M. synoviae *neuraminidase [8,56], Dušanić et al. unpublished observations]. Its effect on cell viability has, however, not been reported before this study.

This is the first report of mycoplasma-induced apoptosis in chondrocytes. The XTT proliferation assay was performed on chicken chondrocytes infected with *M. synoviae *and a major decrease in cell respiration was noted, suggesting cell death had occurred (Figure 1). Chondrocyte morphology, evaluated under phase contrast microscope and confocal microscope combined with anti-CD44 receptor and propidium iodide staining additionally indicated cytopathology with apoptotic features in chondrocytes infected with *M. synoviae *(Figures 2 and 3).

RT-qPCR analysis, performed on 15 genes involved in apoptosis (Table 2, Figure 4), involved NO production in apoptosis induction. The Griess assay confirmed secretion of NO to supernates (Figure 6). NO production is closely linked to chondrocyte apoptosis and cartilage loss in human arthropathies such as osteoarthritis and rheumatoid arthritis [30-33]. Also, NO production was shown to be strongly upregulated in chicken macrophages infected with *M. synoviae *or in contact with its lipoprotein MSPB [57]. Both endogenously and exogenously produced NO is capable of inducing chondrocyte apoptosis, but the analysis here was limited to NO produced by chondrocytes as a result of *M. synoviae *infection. As expected, *Mapk11 *(encoding p38B, the major inducer of NO-linked apoptosis) was upregulated as well, 24 h and 48 h after infection. It has been reported that p38 causes upregulation and stimulation of caspase 3 activity, and upregulation of NFκB transcription factor [34,35]. In our study, upregulation was noticed in genes encoding both caspase 3 and NFκB1, as well as in genes encoding other related apoptotic proteins (Figures 4 and 5).

Increased transcription of caspase genes is the most probable explanation for the formation of apoptotic bodies and cell shrinkage in infected CCH [25]. Although genes encoding endonuclease G, which is released from the mitochondria during apoptosis, and Bak1, a pro-apoptotic mitochondrial protein, were not upregulated, another mitochondrial nuclease, encoded by *AIFM1*, was strongly upregulated 72 h after infection, indicating mitochondrial involvement (Figures 4 and 5). Occurrence of Fas receptor mediated apoptosis, which has been reported in chondrocytes [35], was disregarded due to unchanged transcription of both *Fas *and *FASLG *during CCH infection with *M. synoviae *(Additional file 1, Figure S1). Apoptosis inhibitor, encoded by *BCL2*, was slightly upregulated after 72 h, but so was *htrA3*, encoding the inhibitor of Bcl2. The expression of another apoptotic inhibitor, encoded by *XIAP*, remained unaltered (Additional file 1, Figure S1).

Taken together, our results indicate that *M. synoviae *induces endogenic nitric oxide mediated, caspase 3 and 8 dependent apoptosis in chicken chondrocytes, which involves a loss of mitochondrial function (Figure 5).

5-fluorouracil was used as a positive control of the experiment due to its well established apoptotic effect on other cell lines [58]. Compared to *M. synoviae*, it induced a stronger and faster apoptotic response in CCH (Figure 4, Additional file 1, Figure S1, Figures 2 and 3). The profile of gene expression modulations indicate a Fas receptor mediated, caspase 3 independent cell death involving mitochondrial inactivation. Interestingly, the Jurkat cell line that was used as a positive control of the viability test shows great susceptibility to *M. synoviae *infection (Figure 1).

Although live intracellular *M. synoviae *were re-cultivated in our previous study 24 and 48 h after infection, the percentage of CCH invasion was relatively low (1.2 ± 0.3 for the type strain WVU 1853) [8]. This study demonstrates a high susceptibility to cell death in infected chondrocytes. This suggests that *M. synoviae *affects CCH both from the outer side of the membrane, probably in a Fas independent manner, and from its location within the cell. It is also possible that mycoplasmas die in cell culture due to their complex nutritional demands, but their antigens continue to stimulate CCH and cause stress. An example of macrophage stimulation to produce NO, IL6 and IL1β by *M. synoviae *lipoprotein MSPB has been reported previously [57].

With the exception of caspase 3, patterns of apoptotic gene upregulation were not confirmed on the protein level due to the scarcity of the appropriate specific antibodies to chicken proteins or enzymes involved in apoptotic signal transduction cascades. Even with caspase 3, the antibody used was not specific enough to recognize the cleaved form of the protein (Additional file 2, Figure S2). Still, correlations between demonstrated cytopathologic changes, results of the XTT tests, Griess assay and gene expression data indicate apoptosis and not necrosis as the main mechanism of cell death. As an additional confirmation, CCH infected with *M. synoviae *were labeled using Annexin V-FITC (AV) and propidium iodide (PI) to discriminate between live (AV^-^PI^-^), early apoptotic (AV^+^PI^-^), late apoptotic (AV^+^PI^+^) and primary/secondary necrotic cells (AV^-^PI^+^). The results show an increase in the percentage of cells with phosphatidylserine exposed on the outer membrane layer, to which AV is bound, in the first two days of infection. This was followed by an increase in cells labeled only with PI, indicating secondary necrosis (Additional file 3, Figure S3).

In conclusion, this study provides the first demonstration of mycoplasma-induced apoptosis of chondrocytes. This process may lead to the development of *M. synoviae*-induced infectious synovitis in the chicken, a disease that in many factors resembles human infectious arthritis. It also supports the hypothesis that mycoplasma-induced arthritic conditions in animals might be useful models for understanding the role of mycoplasmas in similar human diseases. Hence, the study may have value outside the immediate interest of avian arthritis.

## List of abbreviations used

NO: nitric oxide; CCH: chicken chondrocytes; XTT: 2, 3-bis-(2-methoxy-4-nitro-5-sulfophenyl)-2H-tetrazolium-5-carboxanilide; RT-qPCR: quantitative real-time polymerase chain reaction; 5-FU: 5-fluorouracil; *Casp3*: gene encoding caspase 3; *BCL2*: gene encoding B-cell CLL/lymphoma 2 protein (Bcl2); *Bak1*: gene encoding Bcl2 antagonist/killer protein 1 (Bak1); *FASLG*: gene encoding the Fas receptor ligand; *tp53*: tumor protein 53 gene, encoding p53 protein; *AIFM1*: apoptosis-inducing factor gene, encoding AIFM1 protein; *CASP8*: gene encoding caspase 8; *endog*: gene encoding endonuclease G; *XIAP*: gene encoding the X-linked inhibitor of apoptosis (XIAP); *NOS2*: gene encoding the inducible nitric oxide synthase (NOS2); *NFκB1*: gene encoding the nuclear factor of kappa light polypeptide gene enhancer in B-cells 1 (NFκB1); *htrA3*: gene encoding the HtrA serine peptidase 3; *Mapk11*: gene encoding the mitogen activated protein kinase 3 (MAPK11 or p38B); *GAPDH*: gene encoding glyceraldehyde-3-phosphate dehydrogenase (GAPDH); Bax: Bcl2-associated X protein; Bid: BH3 interacting domain death agonist (truncated form: tBid).

## Competing interests

The authors declare that they have no competing interests.

## Authors' contributions

DD conceived the study, carried out the isolation of CCH, infection of cells, RNA isolation and subsequent gene expression analysis, all microscopy and NO concentration assays, performed the statistical analysis and drafted the manuscript. DB carried out *M. synoviae *cultivation and CFU determination and participated in manuscript editing. IO advised DD on RT-qPCR and participated in manuscript drafting. IC participated in *M. synoviae *cultivation, determining the number of CFU and performing RT-qPCR. MB participated in performing confocal microscopy and flow cytometry analysis. MN participated in designing the study, drafting the manuscript and coordination between authors. All authors read and approved the final manuscript.

## Supplementary Material

Additional file 1 **Figure S1: Expression of genes for which no significant alterations could be detected after exposure of CCH to *M. synoviae *WVU 1853**. Exposure agent and time of exposure (in hours) are indicated below columns. Control (CTRL) represents in all graphs non-exposed CCH. Results are given as mean values ± standard error for three independent cell treatment experiments with three RT-qPCR replicates for each experiment. (file format: EPS). Click here for file

Additional File 2 **Figure S2: Immunodetection of caspase 3 protein in CCH cell lysates**. CCH were infected with *M. synoviae *WVU 1853 for 24, 48 and 72 h. Cells were then lysed in IP lysis buffer (10 mM Tris HCl pH 7.4, 150 mM NaCl, 0.1% NP-40, 0.002 M EDTA) with 0.1% protease inhibitor cocktail (Sigma) and kept at -20°C. Protein concentration was determined by adding 160 μL of Bradford reagent (Bio-Rad) to 40 μL of cell lysate samples diluted 1:100 in miliQ water and comparing A_595 _values to those obtained for bovine serum albumin (BSA) solutions with known concentrations. Cell lysates containing 60 μg of total proteins were prepared for polyacrilamide gel electrophoresis by adding 2 μL of dithiothreitol (DTT; RD Systems) and 5 μL of loading buffer (Fermentas) to 18 μL of sample. Samples were separated on a 12% polyacrylamide gel (30% acrylamide mix, ammonium persulfate, N, N, N', N'-tetramethylethylenediamine, Trizma base from Sigma-Aldrich, sodium dodecyl-sulfate from Merck) and transferred onto a polyvinyl-difluoride membrane (Imobilon-P, Millipore) in electroblotting buffer (N-cyclohexyl-3-aminopropanesulfonic acid, MetOH from Sigma-Aldrich) by applying 0.8 mA/cm^2 ^of gel for 45 min. The membrane was blocked overnight in 3% BSA at 4°C and incubated in rabbit monoclonal antibodies to human caspase 3 or α/β tubulin (1:2000 in Tween20 solution in phosphate buffered saline pH 7.0 (Tween-PBS), both from Cell Signaling Technology, USA) for 2 h at room temperature. Following washing in 0.05% Tween-PBS, horseradish peroxidase-labeled goat anti rabbit IgG (1:5000 in 0.05% Tween-PBS, Sigma-Aldrich) were used as secondary antibodies. BM chemiluminiscence blotting substrate (Roche) was used for detection on film paper (reagents from Ilford). Note that increased synthesis of caspase 3 is evident, although the antibody failed to recognize 17 kDa fragments of cleaved enzyme. Click here for file

Additional file 3 **Figure S3: Flow cytometry analysis of Annexin V-FITC and propidium iodide stained non-infected CCH (a, b, c, g) and CCH infected with *M. synoviae *WVU 1853 (d, e, f, g)**. CCH were infected as described in the Materials and methods section, washed in PBS and scraped using a rubber policeman. Washed cells were resuspended in 100 μL 1 × binding buffer (10 × stock solution: 0.1 M HEPES pH 7.4, 1.4 M NaCl, 25 mM CaCl_2_) and stained by adding 5 μL Anexin V-FITC (BD Pharmingen) and 10 μL propidium iodide (PI, 10 μg/mL stock, Sigma-Aldrich). Cells were incubated at room temperature for 15 min, followed by addition of another 400 μL of 1 × binding buffer. In order to stain nuclei of live cells, DAPI (final concentration 3 μM, Invitrogen) was used just before flow cytometric analysis. Single color controls for Annexin V-FITC, PI and DAPI were used to set compensations. Annexin V-FITC was detected in the 536/40 nm channel after excitation with the 488 nm blue laser (50 mW). PI was detected in the 675/25 nm after excitation with the 488 nm blue laser (50 mW). DAPI was detected in the 455/25 nm channel after excitation with the 405 nm violet diode laser (100 mW). With this set up no compensation was needed. Cells were analyzed on a CyFlow Space (Partec) fitted with a 488 nm blue laser and violet diode 405 nm with FlowMax software. DAPI positive and CCH positive (based on FSC/SSC) signals were used for gating. Live cells were defined as AV^-^PI^-^, early apoptotic as AV^+^PI^-^, late apoptotic as AV^+^PI^+^, and primary/secondary necrotic cells as AV^-^PI^+^. Click here for file
