# Supplementary figures and images for: Mycoplasma synoviae induces upregulation of apoptotic genes, secretion of nitric oxide and appearance of an apoptotic phenotype in infected chicken chondrocytes
Source: Vet Res. 2012 Jan 26;43(1):7. doi: 10.1186/1297-9716-43-7 (PMC3293721; doi:10.1186/1297-9716-43-7)

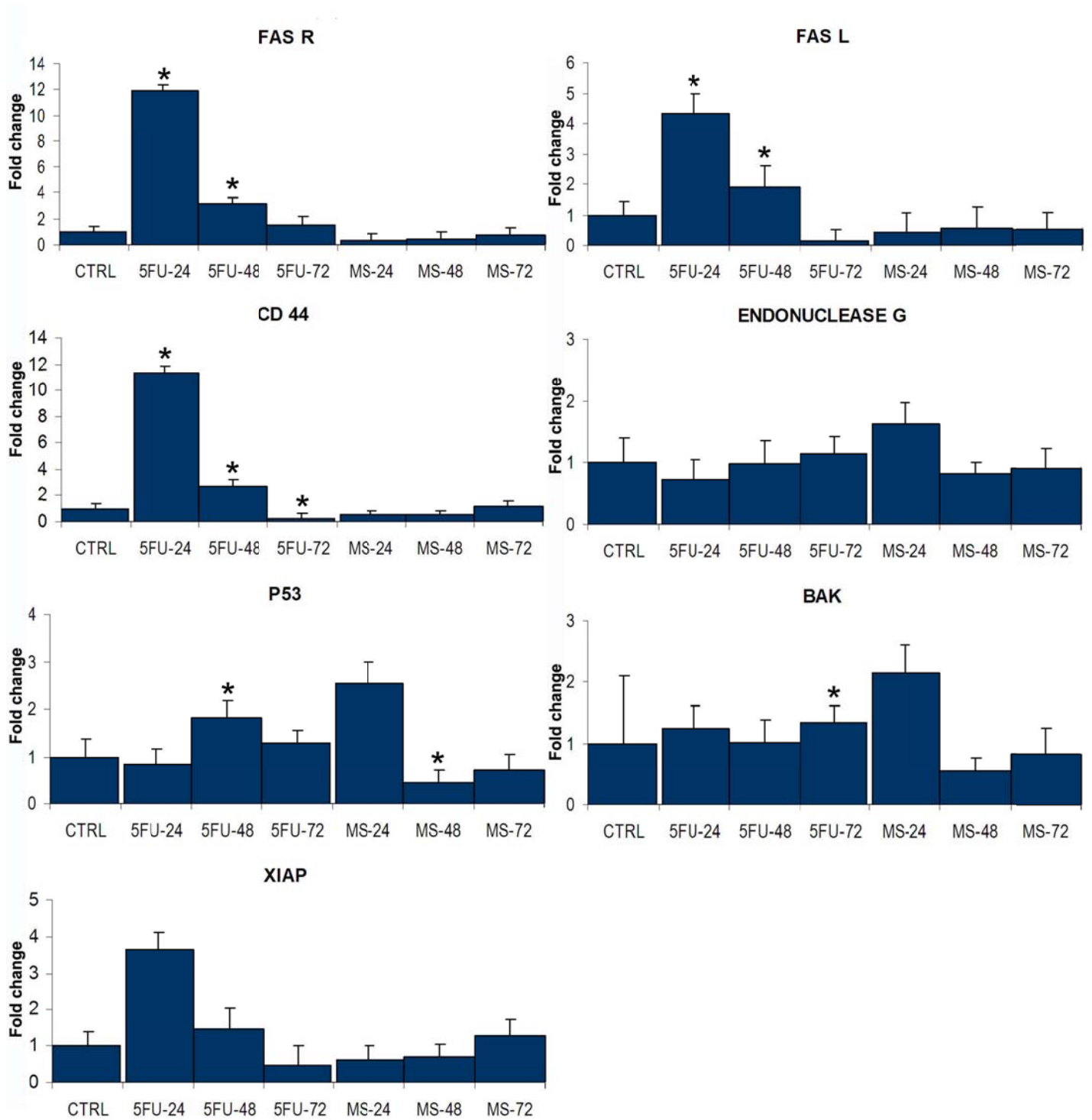

Supplement: Additional file 1 — Figure S1: Expression of genes for which no significant alterations could be detected after exposure of CCH to M. synoviae WVU 1853. Exposure agent and time of exposure (in hours) are indicated below columns. Control (CTRL) represents in all graphs non-exposed CCH. Results are given as mean values ± standard error for three independent cell treatment experiments with three RT-qPCR replicates for each experiment. (file format: EPS). [file 1297-9716-43-7-S1.PDF]

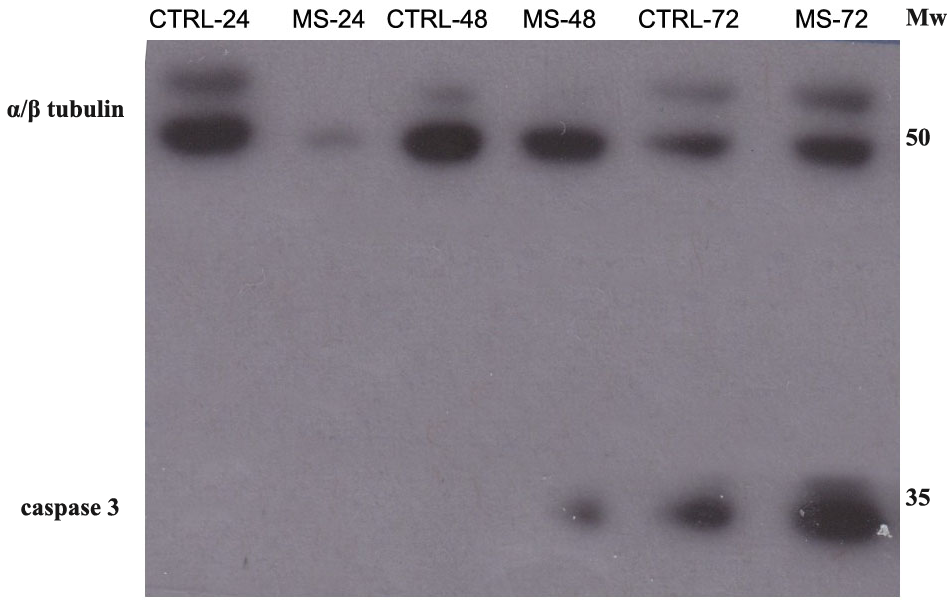

Supplement: Additional File 2 — Figure S2: Immunodetection of caspase 3 protein in CCH cell lysates. CCH were infected with M. synoviae WVU 1853 for 24, 48 and 72 h. Cells were then lysed in IP lysis buffer (10 mM Tris HCl pH 7.4, 150 mM NaCl, 0.1% NP-40, 0.002 M EDTA) with 0.1% protease inhibitor cocktail (Sigma) and kept at -20°C. Protein concentration was determined by adding 160 μL of Bradford reagent (Bio-Rad) to 40 μL of cell lysate samples diluted 1:100 in miliQ water and comparing A595 values to those obtained for bovine serum albumin (BSA) solutions with known concentrations. Cell lysates containing 60 μg of total proteins were prepared for polyacrilamide gel electrophoresis by adding 2 μL of dithiothreitol (DTT; RD Systems) and 5 μL of loading buffer (Fermentas) to 18 μL of sample. Samples were separated on a 12% polyacrylamide gel (30% acrylamide mix, ammonium persulfate, N, N, N', N'-tetramethylethylenediamine, Trizma base from Sigma-Aldrich, sodium dodecyl-sulfate from Merck) and transferred onto a polyvinyl-difluoride membrane (Imobilon-P, Millipore) in electroblotting buffer (N-cyclohexyl-3-aminopropanesulfonic acid, MetOH from Sigma-Aldrich) by applying 0.8 mA/cm2 of gel for 45 min. The membrane was blocked overnight in 3% BSA at 4°C and incubated in rabbit monoclonal antibodies to human caspase 3 or α/β tubulin (1:2000 in Tween20 solution in phosphate buffered saline pH 7.0 (Tween-PBS), both from Cell Signaling Technology, USA) for 2 h at room temperature. Following washing in 0.05% Tween-PBS, horseradish peroxidase-labeled goat anti rabbit IgG (1:5000 in 0.05% Tween-PBS, Sigma-Aldrich) were used as secondary antibodies. BM chemiluminiscence blotting substrate (Roche) was used for detection on film paper (reagents from Ilford). Note that increased synthesis of caspase 3 is evident, although the antibody failed to recognize 17 kDa fragments of cleaved enzyme. [file 1297-9716-43-7-S2.PNG]

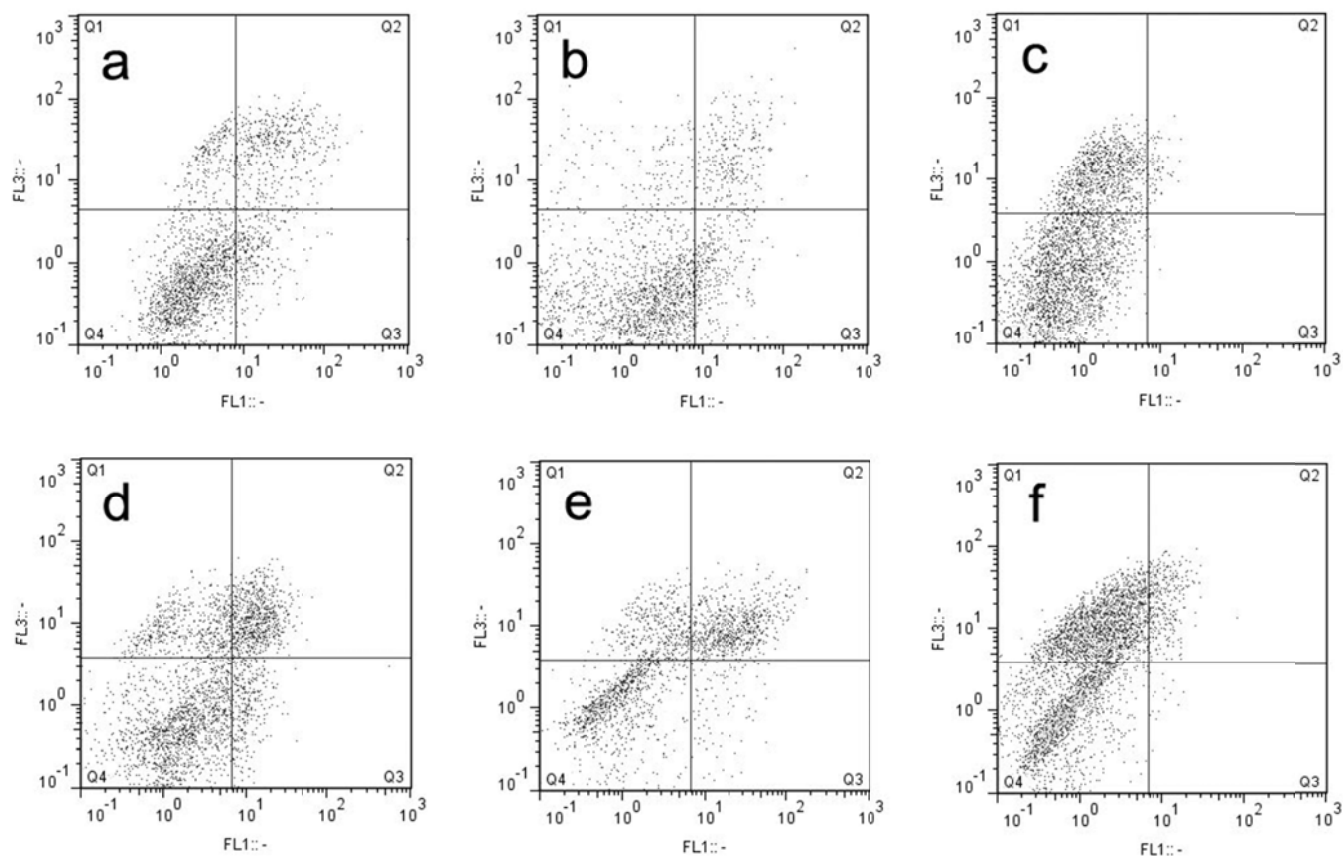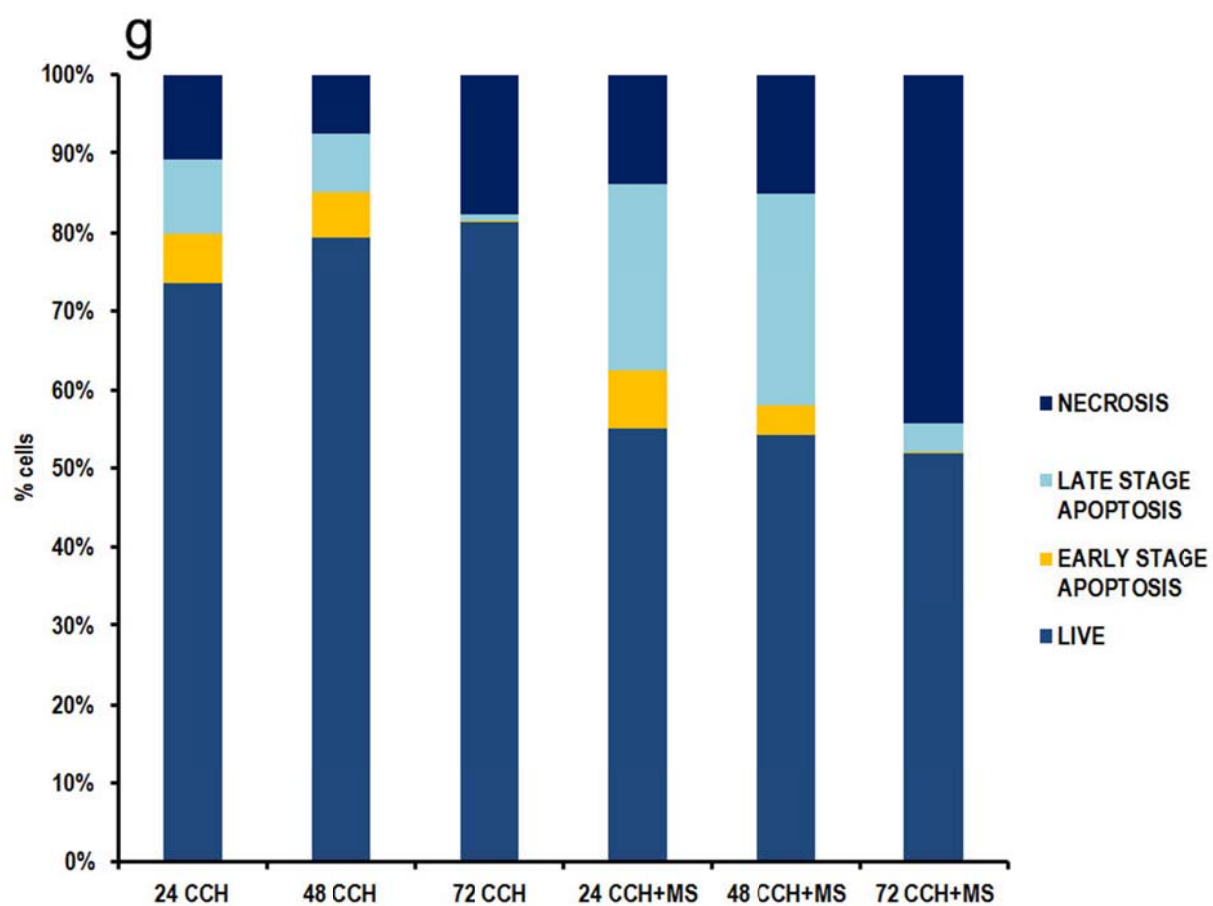

Supplement: Additional file 3 — Figure S3: Flow cytometry analysis of Annexin V-FITC and propidium iodide stained non-infected CCH (a, b, c, g) and CCH infected with M. synoviae WVU 1853 (d, e, f, g). CCH were infected as described in the Materials and methods section, washed in PBS and scraped using a rubber policeman. Washed cells were resuspended in 100 μL 1 × binding buffer (10 × stock solution: 0.1 M HEPES pH 7.4, 1.4 M NaCl, 25 mM CaCl2) and stained by adding 5 μL Anexin V-FITC (BD Pharmingen) and 10 μL propidium iodide (PI, 10 μg/mL stock, Sigma-Aldrich). Cells were incubated at room temperature for 15 min, followed by addition of another 400 μL of 1 × binding buffer. In order to stain nuclei of live cells, DAPI (final concentration 3 μM, Invitrogen) was used just before flow cytometric analysis. Single color controls for Annexin V-FITC, PI and DAPI were used to set compensations. Annexin V-FITC was detected in the 536/40 nm channel after excitation with the 488 nm blue laser (50 mW). PI was detected in the 675/25 nm after excitation with the 488 nm blue laser (50 mW). DAPI was detected in the 455/25 nm channel after excitation with the 405 nm violet diode laser (100 mW). With this set up no compensation was needed. Cells were analyzed on a CyFlow Space (Partec) fitted with a 488 nm blue laser and violet diode 405 nm with FlowMax software. DAPI positive and CCH positive (based on FSC/SSC) signals were used for gating. Live cells were defined as AV-PI-, early apoptotic as AV+PI-, late apoptotic as AV+PI+, and primary/secondary necrotic cells as AV-PI+. [file 1297-9716-43-7-S3.PDF]
